# Efficacy of Immunotherapy in Second-Line Treatment of *KRAS*-Mutated Patients with Non-Small-Cell Lung Cancer—Data from Daily Practice

**DOI:** 10.3390/curroncol30010037

**Published:** 2022-12-29

**Authors:** Magdalena Knetki-Wróblewska, Sylwia Tabor, Adam Płużański, Zofia Lewandowska, Andrzej Tysarowski, Hubert Pawlik, Dariusz M. Kowalski, Maciej Krzakowski

**Affiliations:** 1 Department of Lung Cancer and Thoracic Tumours, The Maria Sklodowska-Curie National Research Institute of Oncology, 02-781 Warsaw, Poland; 2 Cancer Molecular and Genetic Diagnostics Department, The Maria Sklodowska-Curie National Research Institute of Oncology, 02-781 Warsaw, Poland; 3 Computational Oncology Department, The Maria Sklodowska-Curie National Research Institute of Oncology, 02-781 Warsaw, Poland

**Keywords:** NSCLC, *KRAS* gene, immunotherapy, nivolumab, atezolizumab

## Abstract

Background The implementation of next-generation sequencing (NGS) into daily practice allows for the identification of a greater number of molecular abnormalities. We aimed to confirm the benefits of immunotherapy in the group of patients with *KRAS* aberrations treated within clinical practice. Methods This study was a retrospective analysis of the patients (pts) treated in routine practice within the National Drug Programme in Poland. The NGS was performed using a FusionPlex Comprehensive Thyroid and Lung (CTL) kit (ArcherDx) and sequenced using a MiniSeq (Illumina). The analyses were performed with the R language environment, version 4.1.3. Results A total of 96 pts with chemotherapy-pre-treated advanced NSCLC (CS III–IV) were qualified for nivolumab or atezolizumab treatment following a molecular diagnosis by the NGS and the exclusion of *EGFR* and *ALK* gene abnormalities. A mutation in the *KRAS* gene was found in 26 patients (27%); among them, the variant p.Gly12Cyc (G12C) was the most common (42%). The median PFS and OS for the overall population were 2 months (95% CI: 1.8–2.75) and 10 months (95% CI: 6.9–16.2), respectively. No differences were observed in terms of the mPFS between the *KRAS*-mutated and *KRAS* wild-type (WT) patients. A trend toward a longer OS was observed in the group of patients with the *KRAS* mutation, but the difference was not statistically significant (*p* = 0.43). In the multivariate analysis, the presence of mutations in the KRAS gene had no prognostic significance, while the occurence of grade 3 toxicity and the neutrophil-to-lymphocyte ratio (NLR) > 3.5 were found as statistically significant factors. Conclusions Immunotherapy in the second-line treatment of advanced NSCLC allows for a benefit regardless of the *KRAS* gene mutation status. The treatment sequence, including molecularly targeted drugs such as sotorasib and adagrasib, is still discussed. The NGS is a valuable method to identify a variety of molecular abnormalities in patients with NSCLC in daily practice.

## 1. Introduction

For several years, immune checkpoint inhibitors have been established as the standard of care in patients diagnosed with non-small-cell lung carcinoma (NSCLC) with progression following platinum-based chemotherapy. Nivolumab and atezolizumab trials enrolled patients with the most common molecular abnormalities in the *EGFR* and *ALK* genes.

*EGFR* gene mutations were found in 15% of participants in the CheckMate 057 study. Nivolumab was not superior over docetaxel in this subgroup of patients (HR 1.18; 0.69–2.0) [1]. The data published by other authors confirmed these observations [2,3]. A multivariate analysis of 613 patients, 15% of whom had the *EGFR* gene mutation and 2% *ALK* alterations, showed a negative prognostic value of the *EGFR* mutations and *ALK* rearrangement for progression-free survival (PFS) (HR 1.45; 1.12–1.186) [3]. In recent years, new molecular abnormalities have been found in patients diagnosed with NSCLC, most of them in the *KRAS*, *BRAF*, *MET* and *RET* genes. Mutations in the *KRAS* gene are found in 25–35% of patients diagnosed with NSCLC, with the rates higher in those with adenocarcinoma and in smokers [4,5]. Several mutation variants have been identified, with p.Gly12Cys (approximately 30% of patients) and p.Gly12Asp (G12D) (approximately 23%) being the most common [4,5].

For decades, mutations in the *KRAS* proto-oncogene were considered an unrealistic molecular target for effective targeted therapy. This was due to the properties of the *KRAS*-encoded protein, guanosine triphosphatase (GTPase), which, via extracellular stimulation, contributes to cell cycle regulation by cycling between the active guanosine triphosphatase (GTP)-bound state and the inactive guanosine diphosphate (GDP)-bound state. The active form of the KRAS protein serves as a switch, activating downstream signaling pathways in cells, leading to their growth and proliferation. *KRAS* gene mutations, including the p.Gly12Cys variant most commonly found in NSCLC, result in abnormally high concentrations of the active form of KRAS, leading to the dysregulation of the cell cycle [6].

Recently, there have been new reports from studies evaluating the efficacy of sotorasib and adagrasib, two drugs targeting the *KRAS* p.Gly12Cys mutation. Sotorasib is the first specific and irreversible inhibitor of *KRAS* p.Gly12Cys whose efficacy was confirmed in multi-center clinical trials. Sotorasib covalently binds to a region only present in the inactive, GDP-bound conformation and promotes it while inhibiting oncogenesis. An objective response rate (ORR) was confirmed in 37.1% of patients who had undergone at least one line of systemic therapy. The mPFS was 6.8 months, and the median of the overall survival (OS) reached 12.5 months. Treatment-related serious adverse events, including interstitial pneumonitis, diarrhea and the increased activity of aminotransferases, occurred in 20.6% of patients [7]. The preliminary results of the phase III CodeBreaK 200 study that randomly compared the *KRAS* p.Gly12Cys inhibitor sotorasib versus docetaxel in pre-treated patients were presented at the European Society for Medical Oncology (ESMO) Congress Presidential Symposium. The median PFS in the experimental arm was 5.6 vs. 4.5 months in the control arm, with an mOS of 10.5 and 11.3 months, respectively; the 12-month PFS rate was 24.8% for sotorasib and 10.1% for docetaxel. An improved mPFS was also observed in patients with a history of central nervous system (CNS) metastases [8].

Adagrasib is another covalent *KRAS* p.Gly12Cys inhibitor with a similar mechanism of action to sotorasib but a significantly higher bioavailability. Its efficacy was evaluated in a phase I/II single-arm study in patients pre-treated with standard chemotherapy and immunotherapy. In the study, an ORR was achieved by 42.9% of the patients treated with adagrasib, with the mean response duration of 8.5 months. At 15.6 months of follow-up, the mPFS was 6.5 months, while the mOS was 12.6 months. Grade 3 and 4 adverse events occurred in 44.8% of the patients [9].

Currently, the access to treatments aimed at this molecular target remains limited in many countries, and even if they are available, most patients are still being referred for systemic therapy with PD-1/PD-L1 inhibitors. Considering the incidence of *KRAS* aberrations, the assessment of the efficacy and safety in this population is of primary importance. Real-world data may provide additional insights.

## 2. Methods

### 2.1. Inclusion Criteria

Patients diagnosed with advanced NSCLC (squamous and non-squamous histology), eligible for second-line therapy as part of standard practice, participated in the study. Eligibility criteria included the diagnosis of stage III or IV NSCLC, pre-treatment with one line of palliative chemotherapy, good performance status (ECOG 0–1), measurable lesions detectable by computed tomography (CT), absence of clinically significant autoimmune diseases, absence of *EGFR* and *ALK* molecular abnormalities and normal laboratory parameters. Patients with brain metastases were eligible only in the case of local treatment use and a fixed dose of corticosteroids within 4 weeks before the initiation of immunotherapy. Patients were assigned to nivolumab or atezolizumab at the physician’s discretion. Clinical and pathology data were obtained from available electronic medical records. Prior to the initiation of immunotherapy, patients provided written informed consent for the treatment. The local ethics committee approved the conduct of this analysis.

### 2.2. Treatment Monitoring

A chest CT with contrast, including the upper abdomen (other areas, if clinically indicated), was performed before the start of the immunotherapy. Treatment efficacy was evaluated by CT imaging performed every 3 months or more often if disease progression was clinically suspected. Response to the treatment was assessed according to the Response Evaluation Criteria in Solid Tumours guidelines (RECIST 1.1). Treatment was continued until documented objective disease progression, unacceptable toxicity or death for other reasons. Safety was assessed according to Common Toxicity Criteria for Adverse Events (CTCAE) version 5.0. Overall survival was defined as the time from the initiation of second-line immunotherapy until death. Progression-free survival was defined as the time from the initiation of immunotherapy until disease progression on imaging or clinical examination or death was found, whichever occurred first. Patients alive and without progression at the last observation were censored.

### 2.3. Molecular Diagnostics

Prior to eligibility assessments, patients provided written consent for treatment and, additionally, consent to undergo molecular diagnostics. The method used for molecular diagnostics was NGS. RNA/DNA were extracted from FFPE tissue using the Beckman Coulter Agencourt FormaPure Kit (Brea, CA, USA) and quantified using Quantus (Blue Bell, PA, USA). The libraries for panel sequencing were prepared using FusionPlex (all known and novel ALK, ROS1, RET, MET fusions) and VariantPlex (EGFR, exons 18, 19, 20, 21; KRAS, exons 2, 3, 4 and BRAF, exons 11, 15) Comprehensive Thyroid and Lung Kit (ArcherDx, Boulder, CO, USA). Targeted Next Generation Sequencing—NGS was performed using MiniSeq (Illumina, San Diego, CA, USA). The sequencing data were processed using Archer Analysis (v6.2.1) software which allows for the detection of single nucleotide variants and small indels and fusions. Detected variants have been classified according to Locus Reference Genomic Sequence or NCBI RefSeq: LRG_304t1 (for EGFR), LRG_344t1 (for KRAS), LRG_299t1 (for BRAF), LRG_488t1 (for ALK), LRG_997t1 (for ROS1), LRG_518t1 (for RET), LRG_662t1 (for MET) and named according to HGVS guidelines (varnomen.hgvs.org (accessed on 10 November 2022)).

### 2.4. Pathology Assessments

PD-L1 expression was conducted using formalin-fixed paraffin-embedded tissue samples, stained with PD-L1 (clone 22C3) on Dako platform. PD-L1 expression was evaluated on tumor cells (TC). Eligibility assessment prior to first-line treatment was conducted only if adequate tissue material was available. Documented PD-L1 status was not required for the assessment of eligibility for immunotherapy, the value of which is the focus of the present paper.

All analyses were performed using the R language environment, version 4.1.3, with abundant use of tidy verse, survival and survminer packages. The continuous variables were summarized by the median and interquartile range (IQR), while categorical variables were summarized by count and percentage of the overall number of cases, including by *KRAS*-positive and *KRAS*-negative groups. We used Kaplan–Meier estimator for survival curves, including median survival times and survival at defined time points, and log-rank test to compare survival curves. Univariable and multivariable Cox proportional hazard models were used to estimate hazard ratio (HR). *p*-value < 0.05 was considered statistically significant. All point estimates were reported with a 95% confidence interval (CI). No adjustment for multiple testing was applied.

## 3. Results

### 3.1. Study Group Characteristics

**Figure 1:**
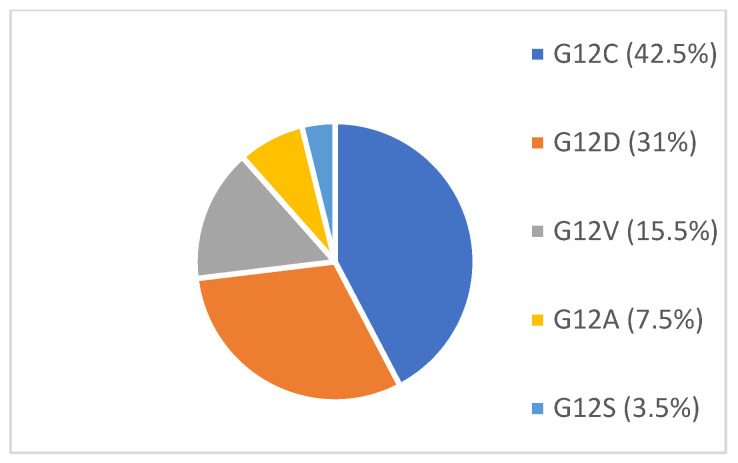
Incidence of *KRAS* molecular aberrations.

The study sample consisted of 96 patients diagnosed with advanced NSCLC and eligible for immunotherapy following a failure of chemotherapy between 2018 and 2021. The characteristics are shown in Table 1. The presence of the *KRAS* molecular aberrations was documented in 26 patients (27%). The most frequent variant was p.Gly12Cys (42%), followed by p.Gly12Asp (30%) and p.Gly12Val (15%). A list of the *KRAS* mutations identified in the study group are shown in Figure 1. There were also other genetic abnormalities identified in the analyzed population, including *RET*—two cases—and one case each of *BRAF p.Val600Glu*, *MAPK*, *CTNNB1* and *MET*. In the *KRAS*-mutant group (compared to the WT patients), there were few cases of liver metastases; a greater proportion of the patients initially had stage 3 disease. No differences were found with respect to other clinical and pathologic features.

### 3.2. Response to Treatment

Most patients (83%) were evaluated for a response to the treatment. In the entire sample, an ORR was observed in 10%, a disease control rate (DCR) in 36% and progressive disease (PD) in 45%. In 17% of the study population, death occurred prior to the first CT assessment. There were no *KRAS* variant-related statistically significant differences in terms of the response to the treatment. The detailed information is presented in Table 2.

### 3.3. Progression-Free Survival

**Figure 2:**
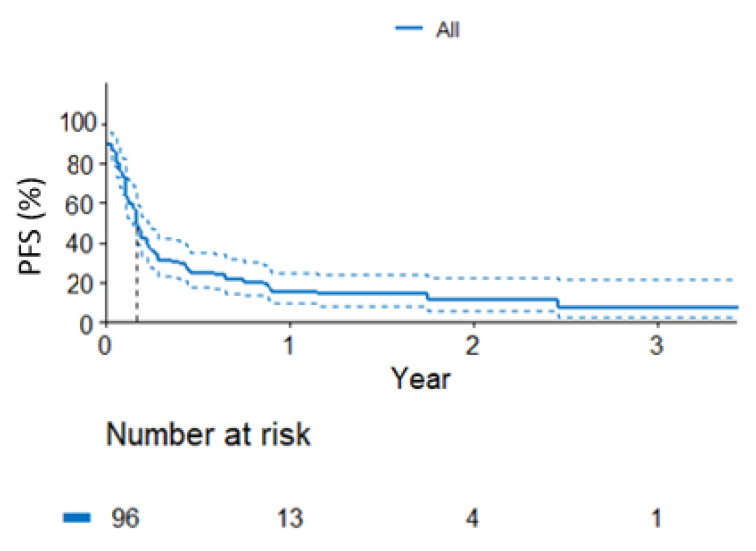
Probability of progression-free survival in the entire population under analysis.

**Figure 3:**
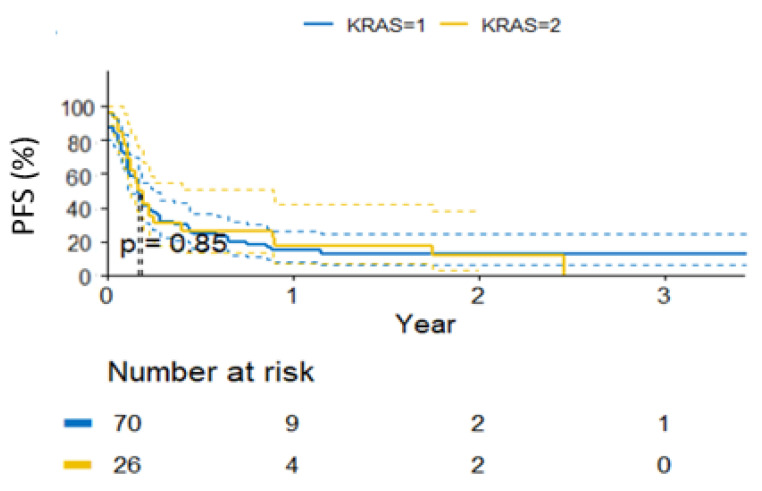
Progression-free survival probability in subgroups by *KRAS* status.

In the entire population, until the data cut-off, PD was observed in 83 patients. The median time to progression was 2 months (95% CI: 1.8–2.75, Figure 2). No *KRAS* status-dependent differences were found in the PFS (*p* = 0.85, Figure 3). The data are shown in Table 3.

### 3.4. Overall Survival

**Figure 4:**
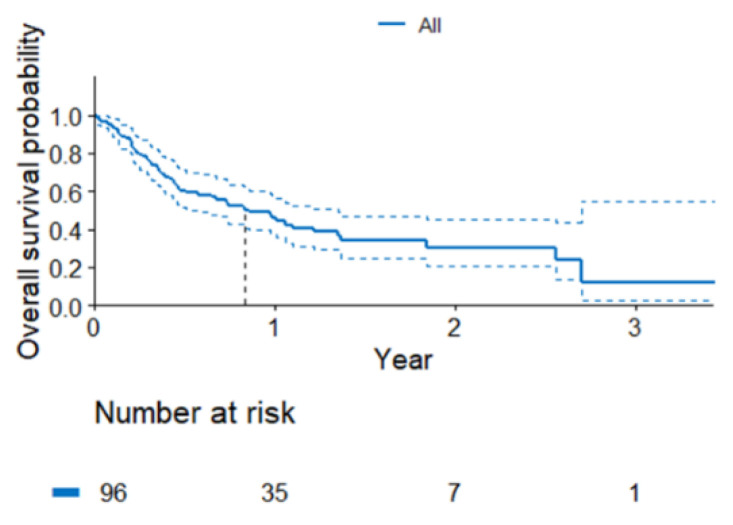
Probability of survival in the entire analyzed group.

**Figure 5:**
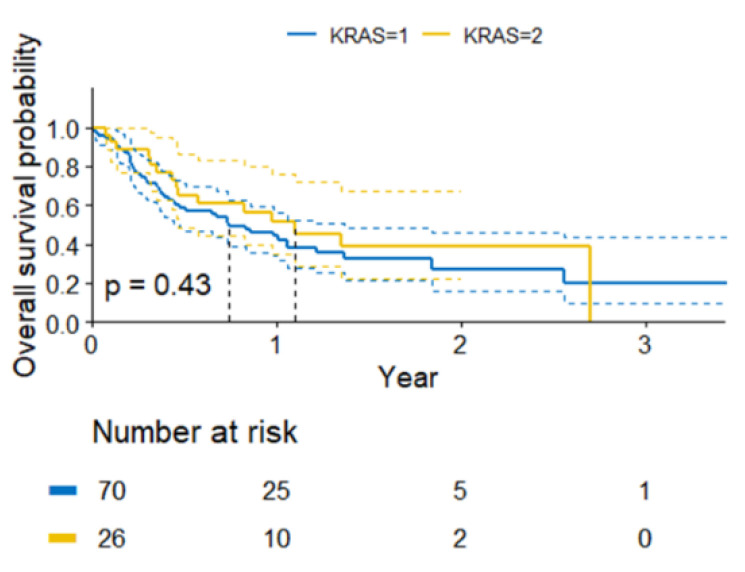
Probability of survival in subgroups by *KRAS* mutation status.

At the time of the analysis, out of the entire analyzed population, 36 patients (37%) remained in the follow-up. The median OS was 10 months (95% CI 6.9–16.2) (Figure 4). No differences in the OS were found between the groups, although there was a trend indicating a longer OS in the patients with *KRAS* gene abnormalities (*p* = 0.43, Figure 5). Due to the small subgroup size, individual *KRAS* mutation variants were not analyzed in detail. The predictive value of the p.Gly12Cys variant vs. other variants was only analyzed. There was no indication of statistically significant differences in the mPFS (*p* = 0.8) and mOS (*p* = 0.38). The survival data are summarized in Table 3.

**Figure 6:**
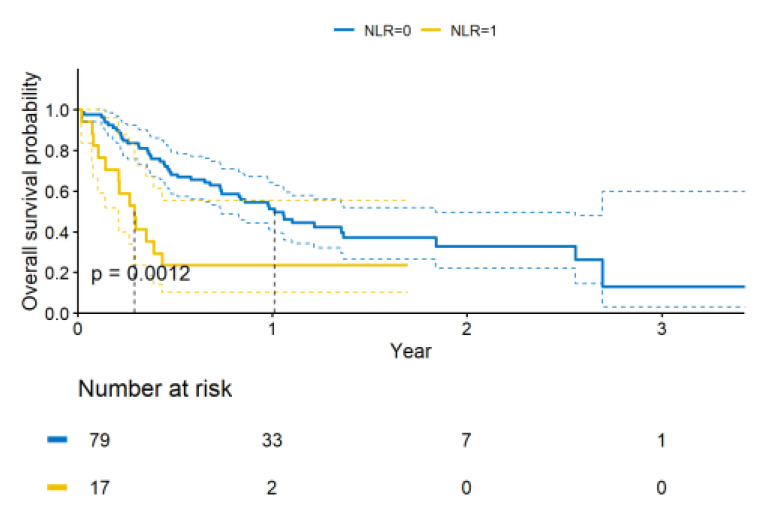
Probability of survival in the entire analyzed group—by NLR.

In addition, we analyzed the prognostic values of selected clinical and laboratory factors, such as the histology, location of metastases, response to previous chemotherapy and NLR. The performance status was not included in the analysis due to the small number of patients with an ECOG performance of 0. The results of the univariate and multivariate analyses are shown in Table 4. The patients with an NLR > 3.5 were at a higher risk of death (Figure 6). The median OS was also shorter if clinically meaningful adverse events occurred. The presence of molecular abnormalities in the *KRAS* gene are not significantly meaningful for a prognosis. Similarly, our analysis found no effects of other factors on the OS.

### 3.5. Safety Profile

In the entire analyzed population, adverse events and abnormal laboratory values were observed in 84% of the patients, including grade 3 events in 13%. The highest rates were observed for anaemia (35% of patients), thyroid dysfunction (30%) and liver enzyme abnormalities (25%), mostly in subjects with liver metastases. There were also cases of respiratory (pneumonitis, 10% of patients) and dermatological (7%) events. Grade 3 adverse events involved the respiratory system (3% of patients) and diarrhea (2%). In addition, there were two cases of a pulmonary embolism and bleeding unrelated to the immunotherapy. No significant differences in the rate of adverse events depending on the *KRAS* gene mutation status were found (Table 5). A detailed analysis of the immunotherapy-related adverse events and practical implications will be presented in a separate publication.

## 4. Discussion

The diagnostic and therapeutic guidelines consider immunochemotherapy to be the first-line treatment of choice in advanced NSCLC; however, in many patients, this type of treatment is not feasible (e.g., when there is no adequate material available to assess the PD-L1 expression) [10,11]. In these cases, a sequential treatment is the treatment of choice: platinum-based chemotherapy followed by second-line immunotherapy (atezolizumab, nivolumab and pembrolizumab). Among the multiple molecular abnormalities documented in patients diagnosed with NSCLC, *KRAS* gene mutations are the most frequent. It is therefore important to determine the value of the available methods of systemic treatment in this population, with particular emphasis on immunotherapy. We report the results of treatment with PD-1 inhibitors in a real-world setting.

Recently, there have been several analyses of patients treated in controlled clinical trials and in a real-world setting.

### 4.1. Efficacy of Immunotherapy in Patients with KRAS Gene Mutations

Our analysis showed that immunotherapy as a second-line treatment provides clinical benefits regardless of the *KRAS* mutation status. However, improved survival, at the level of trend, was found in patients with *KRAS* gene mutations (mOS 13.2 vs. 8.8; *p* = 0.43). A meta-analysis of the first-line treatment in six clinical trials (IMpower-150, Keynote-189, Keynote-042, Oak, Poplar and CheckMate-057) included a total of 1313 patients (386 *KRAS*-mutant and 927 *KRAS* WT tumors) [12]. The population was heterogeneous in terms of the treatment regimens, including both immunotherapy alone as a second-line treatment and immunotherapy combined with chemotherapy or bevacizumab. In the entire analyzed population, the patients with *KRAS*-mutant NSCLCs were found to have improved survival with the use of immunotherapy vs. chemotherapy: OS (0.59 [0.49–0.72]; *p* < 0.00001) and PFS (0.58 [0.43–0.78]; *p* = 0.0003). Overall survival was also found to be longer in the *KRAS*-mutant patients vs. their *KRAS* WT ones (*p* = 0.001). An additional analysis concerning only a supgroup of patients receiving nivolumab or atezolizumab found immunotherapy to be beneficial only in the *KRAS*-mutant subgroup (HR 0.65, 95% CI: 0.44–0.96; *p* = 0.03; the wild-type subgroup HR 0.87; 95% CI: 0.68–1.13; *p* = 0.30) [12].

Jeanson et al. presented the results of the analysis of the treatment outcomes of 282 patients treated with immunotherapy, the majority of whom (88%) received second-line nivolumab. Among them, 162 were found to have *KRAS* gene mutations [13]. There were no significant differences with respect to the responses (OR 1.37; 95% CI 0.71–2.63, *p* = 0.348), PFS (HR 0.93; 95% CI 0.71–1.21, *p* = 0.584) or OS (HR 0.93; 95% CI 0.68–1.29, *p* = 0.682) between the *KRAS*-mutant and WT patients. The *KRAS* mutation subtypes (p.Gly12Ala, p.Gly12Cys, p.Gly12Asp, p.Gly12Val or p.Gly13Cys) had no predictive value. There was a trend of prolonged PFS and OS in the subgroup with *KRAS* gene mutations and a PD-L1 expression > 50% [13].

In the group of 31 *KRAS*-mutant patients reported by Guo et al., 10 patients received immunotherapy alone, while the others were given immunotherapy in combination with chemotherapy or antiangiogenic agents [14]. No *KRAS* status-related significant differences were found in the PFS. The median PFS in the mutant vs. WT subgroups was 11.0 vs. 7.1 months, respectively (*p* = 0.5714).

Uehara et al. presented the results of a retrospective analysis of 78 patients with *KRAS*, *MET*, *FGFR*, *RET*, *BRAF* and *HER2* alterations, who were given first-line immunotherapy alone or in combination with chemotherapy. Among them, 21 patients had mutations in the *KRAS* gene and 13 received immunotherapy combined with chemotherapy while 8 received immunotherapy. The ORR in the *KRAS*-mutant group was 57%, mPFS 16.2 months (95% CI 6.3-NR) while the mOS was 31.3 months (95% CI: 9.0-NR) [15].

A piece of interesting information was provided by a retrospective study using the IMMUNOTARGET registry [16]. It reported the outcomes of 551 patients treated with nivolumab, pembrolizumab, atezolizumab or durvalumab in the first-line (5%), second-line (41%), third-line (26%), fourth-line (13%) or later-lines (14%) setting. The molecular alterations in this population included mutations in *KRAS* (271 patients), *EGFR* (125 patients), *BRAF* (43 patients), *MET* (36 patients), *HER2* (29 patients), *ALK* (23 patients), *RET* (16 patients) and *ROS1* (7 patients). The median PFS in the overall population was 2.8 months, while the mOS was 13.3 months. In the *KRAS*-mutant population, the ORR was achieved in 26% of the patients with an mPFS of 3.2 months. The type of *KRAS* gene alteration had no predictive value [16]. The results of a pooled analysis of data from 12 clinical trials focusing on patients with mutations in the *KRAS* gene were presented at the American Society of Clinical Oncology Congress (ASCO) in 2022 [17]. Patients in the pooled population were given chemotherapy, immunochemotherapy or immunotherapy as the first-line systemic treatment. The analysis included 1430 patients with a known *KRAS* status; aberrations were found in 39% of the cases (including the p.Gly12Cys mutation in 11% of the patients). No differences were found between the *KRAS*-mutant vs. WT patients in terms of the ORR, regardless of the specific treatment method. In the immunotherapy group, there was a trend toward a longer OS in the subgroup with confirmed *KRAS* mutations, but the difference was not statistically significant; the mOS was 22.4 and 18.7 months, respectively (HR 1.12; 95% CI 0.86–1.46). The findings were similar in the chemotherapy group, with an mOS of 17.1 vs. 14.9 months (HR 1.02; 95% CI 0.81–1.29), respectively. In the case of immunotherapy alone, the mOS was 16.2 vs. 16.4 months (HR 1.01; 95% CI 0.76–1.34), respectively.

In the present study, molecular diagnostics were performed using tissue material. In contrast, Sciortino et al. reported on the treatment outcomes in patients whose molecular diagnostics used material from a liquid biopsy assessed by a liquid biopsy Idylla KRAS assay. A total of 22 patients were treated, including second-line treatment with immune checkpoint inhibitors. The prognostic value of the p.Gly12Cys mutation for the PFS was documented in this group. The medians for the mutant vs. WT groups were 23 and 5 months, respectively (HR = 3.28; 95% CI = 0.86–12.5; *p*-value = 0.03). There was no difference in the OS [18].

### 4.2. KRAS Mutation Variant

Due to the small group size, less common *KRAS* mutation variants were not analyzed for prognostic value. The comparison was only between the patients with the p.Gly12Cys variant and those with other variants; there were no differences in the survival parameters. The findings reported by other authors are inconsistent. Arbour et al. analyzed 1194 patients with *KRAS* mutations. The p.Gly12Cys variant was identified in 46% of the patients. The median OS was similar in the G12C group (13.4 months) and the group of patients with other variants (13.1 months; *p* = 0.96) [19]. Other authors reported similar results [13,20]. In contrast, Tamiya analyzed 218 patients treated with second-line immunotherapy and found a significantly longer mPFS in *KRAS* p.Gly12Cys or p.Gly12Val patients compared with other *KRAS* mutations, and at the same time, these *KRAS* mutation variants correlated with high PD-L1 expression [21]. In another study that evaluated immunotherapy in a group of 87 patients, those with the Gly12Asp variant had a higher probability of a therapeutic response (OR (95% CI) = 0.31 (0.09–0.95), *p* < 0.05) and a statistical trend toward improved survival (HR (95% CI) = 0.53 (0.21–1.36), *p* = 0.185) [22].

### 4.3. Other Prognostic Factors

Using data supporting the value of immunotherapy in the second-line setting, it is possible to identify the profile of patients who are more likely to achieve clinical benefit. The key factors include a good performance status (ECOG 0–1), the absence of brain and liver metastases and the long duration of the response to previous systemic therapy [23,24,25,26,27].

Another factor showing a positive correlation with survival parameters is the occurrence of irAEs during immunotherapy [25,26,27,28].

The drug program that sets the eligibility criteria for immunotherapy in Poland only allows for the treatment of patients with a good performance status (ECOG 0–1). For this reason, the benefits of immunotherapy in patients with a worse performance status have not been analyzed. In the population of interest, the course of disease was less favorable in patients with secondary lesions in the liver and CNS, but the differences were nonsignificant. Immunotherapy proved beneficial regardless of the *KRAS* mutation status: there was a trend of improved prognosis in *KRAS*-mutant patients, but the difference was not statistically significant. Among the factors evaluable prior to confirming eligibility for immunotherapy, the NLR proved to be the most outstanding. There have been several other reports of similar findings. In a meta-analysis of 23 trials involving 2068 patients diagnosed with NSCLC and receiving chemotherapy, an elevated NLR correlated with a poorer prognosis for OS (HR = 1.62; 95% CI: 1.41 to 1.87; *p* < 0.001) and PFS (HR = 1.47; 95% CI: 1.25 to 1.72; *p* < 0.001) [29]. This meta-analysis included publications with a heterogeneous set of parameters; the NLR values were between 2.5 and 6. In this paper, we assumed the value of 3.5 (the median for all patients). In the multivariate analysis, that value proved to be an independent negative prognostic factor (HR 2.2; 95% CI 1.1–4.4, *p* = 0.02). Higher NLR values mean an increased neutrophil count and/or a decreased lymphocyte count. Neutrophils may, among other things, stimulate the secretion of inflammatory cytokines, such as interleukin (IL)-1, IL-6 and the tumor necrosis factor (TNF). Low values of circulating lymphocytes may correlate with lower levels of tumor-infiltrating lymphocytes and decreased levels of anti-tumor T cells. This creates an immunosuppressive tumor microenvironment, which may decrease the efficacy of immunotherapy [30].

As we have already mentioned, the safety profile of immunotherapy and irAEs will be analyzed in a separate paper. It should be mentioned here, however, that a complication observed in the population of interest included pulmonary embolism and bleeding unrelated to immunotherapy. These events had a negative impact on the prognosis in the described population. We found no differences in the rates of treatment-related adverse events between the patients with *KRAS* gene aberrations and those with *KRAS* wild type, which is consistent with previous reports [13].

The limitations of this research project include its single-institution character and a relatively small number of patients with confirmed molecular aberrations. Consequently, we were unable to evaluate the predictive value of the mutation variants. Moreover, the retrospective design and limited scope of the NGS panel made it impossible to assess the effects of co-mutations such as STK11 or KEAP1, both of which have preliminary evidence showing their prognostic value in patients receiving immune checkpoint inhibitors [5]. None of our patients in the present analysis received sotorasib or other treatments aimed at this molecular target following the failure of immunotherapy. For this reason, we were unable to determine the value of sequential treatment involving targeted therapy and immunotherapy.

## 5. Conclusions

Immune checkpoint inhibitors are now established as the standard of care in advanced NSCLC, both given alone and in combination with chemotherapy, depending on the PD-L1 expression status. This paper presents a retrospective analysis of the treatment outcomes in 96 patients, 26 of whom were found to have *KRAS* mutations, the most common molecular aberration in the overall NSCLC population. The clinical benefit with immunotherapy as evidenced by the ORR, PFS or OS was achieved regardless of the *KRAS* gene status. There was only a statistical trend for a longer OS in patients with confirmed mutations, but the difference was not significant. There is a need to collect additional data with regard to the efficacy and safety of immunotherapy in patients with *KRAS* gene mutations among bigger groups of patients—most of the published data concerned relatively small populations. Meta-analyses of the data from clinical trials including patients with mutations in the *KRAS* gene are a valuable source of knowledge, but collecting information from daily practice may provide additional and more detailed data [31,32]. The mandatory assessment of the *KRAS* gene status in prospective clinical trials would be helpful to obtain the most valuable data. The optimal treatment sequence is likely to remain contentious until more real-world data and the results of randomized trials are available.

## Figures and Tables

**Table 1:** Characteristics of patients.

Characteristic	*KRAS* (−), *n* = 70 ^1^	*KRAS* (+), *n* = 26 ^1^	*p*-Value ^2^
**Age**	66.81 (7.66)	64.96 (8.03)	0.37
**Gender**			0.71
Female	38 (54%)	13 (50%)	
Male	32 (46%)	13 (50%)	
**Histology**			0.54
Adenocarcinoma	52 (74%)	21 (81%)	
Squamous	10 (14%)	2 (8%)	
Other	8 (12%)	3 (11%)	
**Clinical stage** **(primary)**			0.002
I	-	1 (3.8%)	
II	5 (7.1%)	3 (12%)	
III	5 (7.1%)	8 (31%)	
IV	60 (86%)	14 (54%)	
**ECOG**			0.60
0	4 (5.7%)	-	
1	64 (91%)	26 (100%)	
2	2 (2.9%)	-	
**Smoking history**			>0.99
No	6 (8.6%)	2 (7.7%)	
Yes	64 (91%)	24 (92%)	
**KRAS variant**			>0.99
G12C	-	11 (42%)	
Other	-	15 (58%)	
**PD-L1 expression**			0.82
<1%	21 (58%) **	11 (85%) **	
1–49%	14 (38%) **	2 (15%) **	
No data	34 (48%)	13 (50%)	
**Brain metastases**	17 (24%)	4 (15%)	0.35
**Liver metastases**	22 (31%)	3 (12%)	0.048
**Previous chemotherapy**			0.88
cisplatin/pemetrexed	29 (41%)	10 (38%)	
carboplatin/pemetrexed	9 (13%)	6 (23%)	
carboplatin/ vinorelbine	3 (4.3%)	2 (7.7%)	
Vinorelbine	8 (11%)	1 (3.8%)	
cisplatin/etoposid	3 (4.3%)	-	
cisplatin/ gemcytabine	3 (4.3%)	1 (3.8%)	
cisplatin/vinorelbine	11 (16%)	5 (19%)	
paclitaxel/carboplatin	2 (2.9%)	1 (3.8%)	
**Response to chemotherapy**			0.14
CR	3 (4.3%)	5 (19%)	
PR	13 (19%)	3 (12%)	
SD	27/70 (39%)	10/26 (38%)	
PD	27 (39%)	8 (31%)	
**NLR > 3.5**	12 (17%)	5 (19%)	0.77

^1^ Mean (SD); n/*n* (%); ^2^ Wilcoxon rank sum test; Pearson’s Chi-squared test; Fisher’s exact test. ** among patients with PD-L1 known status, CR—complete response, PR—partial response, SD—stable disease, PD—progressive disease, NLR—neutrophil to lymphocyte ratio.

**Table 2:** Response to treatment by *KRAS* mutation status.

	*KRAS* (−), *n* = 70	*KRAS* (+), *n* = 26	*p*-Value
ORR			0.49
CR	-	1 (3.8%)	
PD	32 (46%)	12 (46%)	
PR	7 (10%)	2 (7.7%)	
SD	20 (28%)	5 (19%)	
No data *	11 (16%)	6 (23%)	

* Death before radiological assessment.

**Table 3:** Survival parameters in the entire analyzed population.

	All Population (*n* = 96)	*KRAS* (+)	*KRAS* (−)	*p*
mPFS	2 (95% CI: 1.8–2.75)	2.16 (95% CI: 1.5–10)	2 (95% CI:1.4–3.2)	0.85
mOS	10 (95% CI 6.9–16.2)	13.2 (95% CI: 5.3–16)	8.8 (95% CI: 5.6-NA)	0.43

Abreviations: mPFS—median progression-free survival; mOS—median overall survival.

**Table 4:** Univariate and multivariate analysis of overall survival for the entire population.

	Univariate Analysis				Multivariate Analysis		
Factor	Level	HR	95% CI		HR	95% CI	*p*-Value
Toxicity	0	Ref.					
	1	1.4	0.6–3.1	0.4049	0.6	0.1–5	0.65
Toxicity G ≥ 3	0	Ref.					
	1	2.3	1.2–4.5	0.0121	2.6	1.2–5.7	0.01
Liver metastases	0	Ref.					
	1	1.5	0.8–2.6	0.2026	1.6	0.8–3.2	0.17
Brain metastases	0	Ref.					
	1	1.1	0.6–2	0.7441	1.1	0.5–2.1	0.88
Histopathology	Non-squamous	Ref.					
	Squamous	0.6	0.3–1.4	0.2349	0.9	0.4–2.4	0.89
NLR	0	Ref.					
	1	2.7	1.5–5.1	0.0012	2.2	1.1–4.4	0.02
*KRAS*	*KRAS(−)*	Ref.					
	*KRAS(+)*	0.8	0.4–1.4	0.427	1	0.5–2	0.94

Abbreviations: NLR (neutrophil-to-lymphocyte ratio), G—grade.

**Table 5:** Rates of treatment-related adverse events in subgroups by *KRAS status*.

	*KRAS* (−), *n* = 70	*KRAS* (+), *n* = 26	*p*-Value
Overall toxicity	58 (83%)	23 (88%)	0.75
Grade > 3 toxicity	8 (14%)	5 (22%)	0.50

## Data Availability

The data that support the findings of this study are available from the corresponding author upon reasonable request.
